# Single-cell RNA-sequencing and microarray analyses to explore the pathological mechanisms of chronic thromboembolic pulmonary hypertension

**DOI:** 10.3389/fcvm.2022.900353

**Published:** 2022-11-10

**Authors:** Ran Miao, Xingbei Dong, Juanni Gong, Yidan Li, Xiaojuan Guo, Jianfeng Wang, Qiang Huang, Ying Wang, Jifeng Li, Suqiao Yang, Tuguang Kuang, Min Liu, Jun Wan, Zhenguo Zhai, Jiuchang Zhong, Yuanhua Yang

**Affiliations:** ^1^Medical Research Center, Beijing Institute of Respiratory Medicine, Beijing Chao-Yang Hospital, Capital Medical University, Beijing, China; ^2^Department of Respiratory and Critical Care Medicine, Beijing Institute of Respiratory Medicine, Beijing Chao-Yang Hospital, Capital Medical University, Beijing, China; ^3^Chinese Academy of Medical Sciences and Peking Union Medical College, Beijing, China; ^4^Department of Echocardiography, Beijing Chao-Yang Hospital, Capital Medical University, Beijing, China; ^5^Department of Radiology, Beijing Chao-Yang Hospital, Capital Medical University, Beijing, China; ^6^Department of Interventional Radiology, Beijing Chao-Yang Hospital, Capital Medical University, Beijing, China; ^7^Department of Pathology, Beijing Chao-Yang Hospital, Capital Medical University, Beijing, China; ^8^Department of Radiology, China-Japan Friendship Hospital, Beijing, China; ^9^Department of Respiration, Beijing Anzhen Hospital, Capital Medical University, Beijing, China; ^10^Department of Pulmonary and Critical Care Medicine, Center of Respiratory Medicine, China-Japan Friendship Hospital, National Clinical Research Center for Respiratory Diseases, Beijing, China; ^11^Heart Center and Beijing Key Laboratory of Hypertension, Beijing Chao-Yang Hospital, Capital Medical University, Beijing, China

**Keywords:** chronic thromboembolic pulmonary hypertension, single-cell RNA-sequencing, microarray, circRNA, miRNA, mRNA

## Abstract

**Objective:**

The present study aimed to explore the pathological mechanisms of chronic thromboembolic pulmonary hypertension (CTEPH) using a gene chip array and single-cell RNA-sequencing (scRNA-seq).

**Materials and methods:**

The mRNA expression profile GSE130391 was downloaded from the Gene Expression Omnibus database. The peripheral blood samples of five CTEPH patients and five healthy controls were used to prepare the Affymetrix microRNA (miRNA) chip and the Agilent circular RNA (circRNA) chip. The pulmonary endarterectomized tissues from five CTEPH patients were analyzed by scRNA-seq. Cells were clustered and annotated, followed by the identification of highly expressed genes. The gene chip data were used to identify disease-related mRNAs and differentially expressed miRNAs and circRNAs. The protein–protein interaction (PPI) network and the circRNA–miRNA–mRNA network were constructed for each cell type.

**Results:**

A total of 11 cell types were identified. Intersection analysis of highly expressed genes in each cell type and differentially expressed mRNAs were performed to obtain disease-related genes in each cell type. TP53, ICAM1, APP, ITGB2, MYC, and ZYX showed the highest degree of connectivity in the PPI network of different types of cells. In addition, the circRNA–miRNA–mRNA network for each cell type was constructed.

**Conclusion:**

For the first time, the key mRNAs, miRNAs, and circRNAs, as well as their possible regulatory relationships, during the progression of CTEPH were analyzed using both gene chip and scRNA-seq data. These findings may contribute to a better understanding of the pathological mechanisms of CTEPH.

## Introduction

Chronic thromboembolic pulmonary hypertension (CTEPH) is a rare small-vessel arteriopathy characterized by persistent pulmonary arterial obstruction that is caused by organized fibrotic thrombi with secondary microvascular remodeling, which may lead to increased vascular resistance, pulmonary hypertension, and heart failure (1). Pulmonary endarterectomy is currently the standard therapy and the only curative treatment for CTEPH, which is associated with a 5-year survival rate of 83% for operable patients (2). However, not all patients with CTEPH are eligible for surgery. Moreover, CTEPH is often diagnosed at an advanced stage due to misdiagnosis or delayed symptoms, resulting in a poor prognosis; the 5-year survival rate of CTEPH patients is less than 40% (3). Therefore, it is of great clinical significance to further explore the pathophysiological mechanisms of CTEPH.

Various genes, cell types, and signal transduction systems are involved in the occurrence and development of CTEPH (4). Gene microarray and sequencing technology have been widely used to analyze intracellular transcription and signaling pathways (5). In addition, several dysregulated mRNAs, microRNAs (miRNAs), and circular RNAs (circRNAs) in CTEPH have been identified by bulk RNA sequencing (RNA-seq) and chip array analyses (6, 7). For example, Gu et al. (6) analyzed pulmonary artery endothelial cells from five CTEPH patients and five donors for lung transplantation (controls) using Affymetrix gene chip analysis and identified 1,614 differentially expressed (DE) genes in CTEPH. Meanwhile, Halliday et al. (8) characterized the molecular and functional features associated with CTEPH using multiple methods, including bulk RNA-seq. Furthermore, Wang et al. (9) performed miRNA microarray analysis and found that the miRNA let-7d plays a crucial role in CTEPH progression. Additionally, our previous analysis using an Agilent circRNA chip showed that hsa_circ_0046159 was significantly upregulated in CTEPH compared with that in normal blood samples (10). Importantly, single-cell RNA-seq (scRNA-seq) is an emerging technique that can reveal the expression profile of individual cells, making it possible to provide an atlas of the single-cell landscape of pulmonary endarterectomized tissues in CTEPH (11, 12). Taken together, bulk RNA-seq and chip array analyses are mainly used to detect the overall gene expression changes in CTEPH, while scRNA-seq can identify different cell clusters and provide the expression profiles of individual cells.

The present study aimed to obtain a more comprehensive understanding of the pathological mechanisms of CTEPH using gene chip array and scRNA-seq analyses. The mRNA expression profile GSE130391 was downloaded from the Gene Expression Omnibus (GEO) database, and the Affymetrix miRNA chip and the Agilent circRNA chip were prepared using the peripheral blood samples from CTEPH patients and healthy controls. In addition, the pulmonary endarterectomized tissues of CTEPH patients were analyzed by scRNA-seq. Then, the circRNA–miRNA–mRNA network was constructed for each cell type. Our data may provide some insights for the development of CTEPH treatment.

## Materials and methods

### Tissue collection and scRNA-seq

Pulmonary endarterectomized tissues were collected from five patients who were diagnosed with CTEPH (13) and underwent a pulmonary endarterectomy between October 2019 and June 2020 at the Beijing Chao-Yang Hospital, Capital Medical University. The baseline characteristics of these patients are shown in Table 1. The patients were treated with one of the following anticoagulants for at least 3 months: warfarin, rivaroxaban, and low-molecular-weight heparin. All treatments were carried out in accordance with the guidelines.

**TABLE 1:** Baseline characteristics of patients with CTEPH.

Characteristic	Value (number of patients)
Female:Male	1:4
Age (years, mean ± SD)	45.00 ± 13.34
BMI (kg/m^2^, mean ± SD)	25.18 ± 1.18
mPAP (mmHg, mean ± SD)	54.40 ± 3.21
PAWP (mmHg, mean ± SD)	9.60 ± 2.51
PVR (dyn.sec/cm^5^, mean ± SD)	1098.60 ± 103.70
WHO FC I–II:WHO FC III–IV	1:4
CI [L/(min⋅m^2^), mean ± SD]	1.78 ± 0.13
Family history of venous thromboembolism	0
Smoking	2
Bed rest over 24 h	0
Other CTEPH risk factors	
Pulmonary embolism	3
Venous thromboembolism	2
Inflammatory bowel disease	0
Splenectomy	0

CTEPH, chronic thromboembolic pulmonary hypertension; BMI, body mass index; mPAP, mean pulmonary arterial pressure; PAWP, pulmonary artery wedge pressure; PVR, pulmonary vascular resistance; WHO FC, World Health Organization function classification; CI, cardiac index; SD, standard deviation.

Tissues samples were then stored in MACS Tissue Storage Solution (Miltenyi Biotec, Bergisch Gladbach, Germany). This study was approved by the Ethics Committee of Beijing Chao-Yang Hospital, Capital Medical University (Approval number: 2019-K-28) and conformed to the principles outlined in the Declaration of Helsinki. The requirement for written informed consent was waived because discarded pulmonary endarterectomized tissues were used in this study. The tissue samples were dissociated to a single-cell suspension and subjected to 10 × Genomics scRNA-seq using the Illumina NovaSeq platform (Illumina Inc., USA).

### Cell clustering

The scRNA-seq data of five pulmonary endarterectomized tissue samples were integrated by Cell Ranger and then filtered by the R package Seurat (14) with the following filtering conditions: gene number > 200; at least one gene expressed in three cells, and mitochondrial gene expression ratio ≤ 20%. Then, all cells were clustered by the Seurat package, and a two-dimensional scatter diagram was displayed using the UMAP method. Marker genes corresponding to each cell cluster were identified using the FindMarkers function in the Seurat package based on differential analysis. The clusters were then annotated with the marker genes to identify the cell type.

### Identification of highly expressed genes

Significantly highly expressed genes in each cell type were identified using the Seurat package (14). The default threshold parameters were set as follows: min.pct = 0.1; only.pos = TRUE; and logfc.threshold = 0.25. Each time, one cell type was assigned as the comparison group to the other cell types. Genes that met all of the following criteria were screened: (1) expressed in 10% of cells in at least one of the two groups; (2) highly expressed in the comparison group; (3) logFC was greater than 0.25.

### Preparation and preprocessing of miRNA and circRNA expression data

Peripheral blood samples were collected from five CTEPH patients who were admitted to the Beijing Chao-Yang Hospital, Capital Medical University, and from five healthy subjects who underwent a routine physical examination at the same hospital between March 2016 and April 2016. This study was approved by the Ethics Committee of Beijing Chao-Yang Hospital, Capital Medical University (Approval number: 2015-7-24-8) and performed in accordance with the principles outlined in the Declaration of Helsinki. The requirement for written informed consent was waived because discarded blood samples were used in this study, while the research involved no risk to the subjects and the waiver did not adversely affect the rights and welfare of the subjects. The total RNA was extracted from the peripheral blood samples using an RNAprep Pure Blood Kit (Tiangen Biotech Co., Ltd., Beijing, China) and prepared for the Affymetrix miRNA chip and Agilent circRNA chip analyses (10).

The miRNA expression profile in the CEL format was preprocessed by Expression Console (version 1.4), including RMA normalization, distinguishing probe signals from background signals, and integrating probe signals into probe set signals. The circRNA expression profile was preprocessed using the Feature Extraction package, and the chip data were normalized by GeneSpring GX. Two probes (CBC1 and CBC2) with different lengths were used to detect one circRNA; therefore, the detection data of the two probes were mutually verified, and the accuracy of the results was improved.

### Identification and preprocessing of the gene expression profile

The gene expression profiles of both patients and healthy controls were searched in the GEO database, with ‘chronic thromboembolic pulmonary hypertension’ as the keyword. The GSE130391 dataset (8), consisting of 14 CTEPH pulmonary artery samples and 4 control pulmonary artery samples, was finally included in this study. The GPL10558 Illumina HumanHT-12 V4.0 Expression Beadchip platform was used.

The Series Matrix File was downloaded from the GEO database, and the corresponding expression data of CTEPH and control samples were extracted. After processing of the log(2) signal intensity with the Affymetrix Microarray Suite (version MAS 5.0) (15), the probe ID was converted into the gene symbol. Probes that did not correspond to the gene symbol were removed. For different probes mapped to the same gene, the mean value of the probes was taken as the final gene expression value.

### Identification of differentially expressed mRNAs, miRNAs, and circRNAs

Differentially expressed mRNAs, miRNAs, and circRNAs between the CTEPH and control groups were identified using the empirical Bayes t-test provided by the R package limma (version 3.40.6) (16). The thresholds were set at *p* < 0.05 and |log fold change (FC)| > 0.5. CircRNAs that were identified as DE circRNAs by both probes were used for further analysis.

### Identification of disease-related genes

A Venn diagram of gene intersection was developed using significantly highly expressed genes in each cell type and DE mRNAs to obtain disease-related mRNAs in single cells.

### Construction of the protein–protein interaction network

The STRING database (17) was used to predict the interactions between DE genes. The input gene sets were disease-related genes in each cell type, and the species was *Homo sapiens*. The PPI score was set to 0.4 (medium confidence). After obtaining the PPI pairs, Cytoscape software (version 3.4.0) (18) was used to construct the network. The CytoNCA plug-in (version 2.1.6) (19) was used to analyze the degree of connectivity of the node, and the parameter was set without weight. The proteins with a higher degree of connectivity were obtained and named hub proteins.

### Prediction of drugs for disease-related mRNAs in single cells

Based on the disease-related mRNAs in each cell type, drug-gene interactions were predicted using the online drug-gene interaction database (20). The default parameters were set as follows: Source Databases, 22; Gene Categories, 43; and Interaction Types, 31. Meanwhile, approved antineoplastic or immunotherapeutic drugs were screened. We mainly focused on the relation pairs with reference support. The drug–gene network was then constructed by Cytoscape software.

### Construction of the single-cell, disease-related circRNA–miRNA–mRNA network

Based on the disease-related mRNAs in each cell type, miRNAs were predicted using the online database mirwalk3.0 (21). The thresholds were set as follows: binding probability, 0.95; and binding site position, 3′-UTR. The miRNAs should appear in either the miRDB or the TargetScan database. After the miRNA–mRNA relation pairs were obtained, intersection analysis with DE miRNAs identified by a previous analysis was performed. Then, the DE miRNA–DE mRNA relation pairs were obtained.

Based on the disease-related miRNAs and circRNAs identified by a previous analysis, the miRNA–circRNA relation pairs were predicted using miranda software (22). A score of > 140 was used as the threshold. The relation pairs with an opposite expression direction of miRNAs and circRNAs were screened as the final circRNA–miRNA relation pairs.

The circRNA–miRNA–mRNA relation regulated by the same miRNA was screened using the miRNA–mRNA relation pairs and the circRNA–miRNA relation pairs. As circRNAs competitively bind to miRNAs to regulate mRNAs, the circRNA–miRNA–mRNA relation with a consistent expression direction of the circRNAs and mRNAs was screened. Finally, the network was constructed, and the degree of connectivity of each node in the network was analyzed.

### Function analysis of the circRNA–miRNA–mRNA network in each cell type

In the circRNA–miRNA–mRNA network of each cell type, the mRNAs were analyzed by Gene Ontology (GO) (23) biological process (BP) and Kyoto Encyclopedia of Genes and Genomes (KEGG) (24) pathway enrichment analyses using the R package clusterProfiler (version 3.8.1) (25). The BP or pathway with an adjusted *p*-value of less than 0.05 was considered statistically significant.

## Results

### Eleven cell types were identified

**FIGURE 1:**
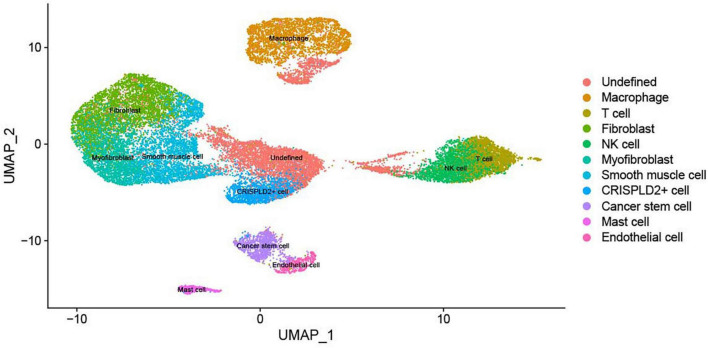
The UMAP plot of 11 cell subtypes in all samples. Different colors indicate different cell types.

The expression matrix of 22,333 genes in 27,140 cells was obtained after scRNA-seq analysis. The cells were then clustered into 17 clusters, with 11 cell types: macrophages, undefined cells, endothelial cells, mast cells, cancer stem cells, CRISPLD2^+^ cells, fibroblasts, myofibroblasts, smooth muscle cells, T cells, and natural killer (NK) cells. The two-dimensional distribution scatter plot of the 11 cell types in all samples is shown in Figure 1.

### Significantly highly expressed genes in each cell type

The genes highly expressed in the 11 cell types were identified using the FindMarkers function in the Seurat package. There were 813, 506, 997, 563, 972, 870, 491, 530, 333, 799, and 726 highly expressed genes in the macrophages, undefined cells, endothelial cells, mast cells, cancer stem cells, CRISPLD2^+^ cells, fibroblasts, myofibroblasts, smooth muscle cells, T cells, and NK cells, respectively.

### Identification of differentially expressed mRNAs, miRNAs, and circRNAs

**FIGURE 2:**
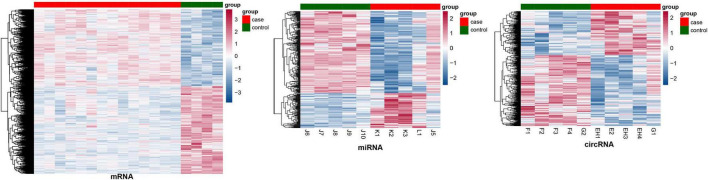
The heatmaps of differentially expressed mRNAs, miRNAs, and circRNAs.

After preprocessing, the expression matrixes of 20,169 mRNAs, 2,578 miRNAs, and 87,935 circRNAs were obtained. Differential expression analysis identified 1,436 DE (670 upregulated and 766 downregulated) mRNAs, 294 DE (88 upregulated and 206 downregulated) miRNAs, and 233 DE (89 upregulated and 144 downregulated) circRNAs. The heatmaps of these DE RNAs are shown in Figure 2.

### Identification of disease-related genes in single cells

**FIGURE 3:**
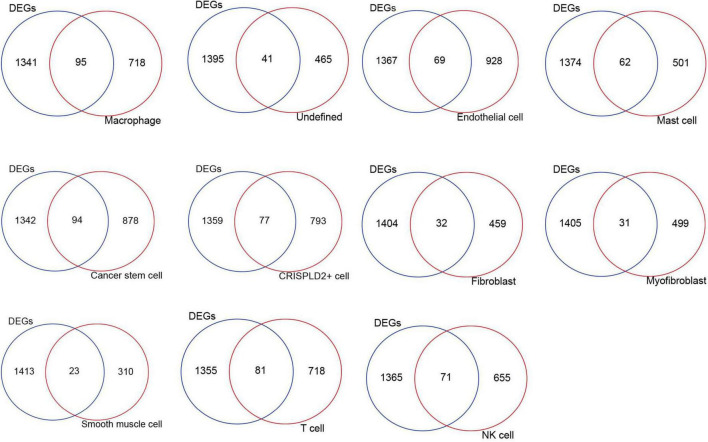
The intersection Venn diagram of differentially expressed mRNAs and highly expressed genes in each cell type.

The intersection analysis of the highly expressed genes in the 11 cell types and DE mRNAs showed that there were 95, 41, 69, 62, 94, 77, 32, 31, 23, 81, and 71 disease-related genes in the macrophages, undefined cells, endothelial cells, mast cells, cancer stem cells, CRISPLD2^+^ cells, fibroblasts, myofibroblasts, smooth muscle cells, T cells, and NK cells, respectively (Figure 3).

### Construction of protein–protein interaction networks based on disease-related genes in single cells

**FIGURE 4:**
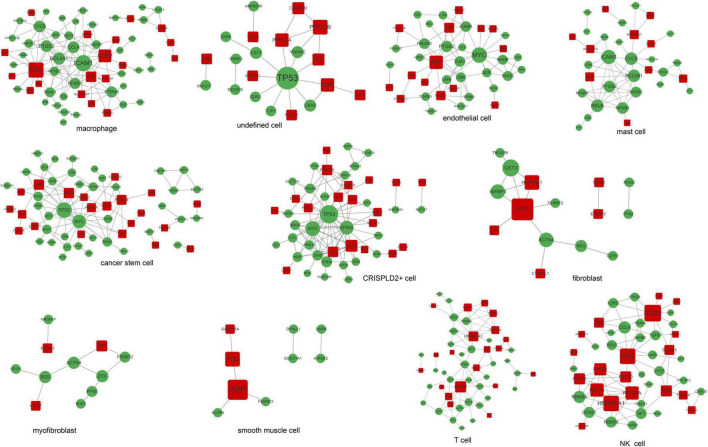
The PPI networks are associated with disease-related genes in each cell type. Red squares represent upregulated mRNAs; green circles represent downregulated mRNAs; gray lines represent protein interactions.

A total of 11 networks were constructed based on the disease-related mRNAs in single cells (Figure 4). The numbers of nodes and relation pairs in each cell type are shown in Table 2. The top 10 genes with a high degree of connectivity are shown in Table 3. TP53 had the highest degree of connectivity in the PPI networks of the cancer stem cells, CRISPLD2^+^ cells, and undefined cells. Intercellular adhesion molecule-1 (ICAM1) had the highest degree in the PPI networks of the macrophages and mast cells. Amyloid beta precursor protein (APP) had the highest degree in the PPI networks of the fibroblasts and smooth muscle cells. Integrin subunit beta 2 (ITGB2) had the highest degree in the PPI networks of the T cells and NK cells. MYC proto-oncogene and bHLH transcription factor (MYC) had the highest degrees in the PPI network of the endothelial cells. Zyxin (ZYX) had the highest degree in the PPI network of the myofibroblasts.

**TABLE 2:** The numbers of nodes and relation pairs (edges) in the PPI network of each cell type.

Cell type	Nodes	Edges
Macrophages	61	112
Undefined cells	21	21
Endothelial cells	39	62
Mast cells	33	53
Cancer stem cells	60	91
CRISPLD2^+^ cells	48	96
Fibroblasts	15	15
Myofibroblasts	11	10
Smooth muscle cells	9	6
T cells	52	82
NK cells	45	78

**TABLE 3:** The top 10 gene nodes in the PPI network of each cell type.

Cell type	Gene node													
Macrophages	ICAM1	FOS	ITGB2	BCL2A1	CYCS	CCL4	PTGS2	PTPN6	NFKB1	SYK				
Undefined cells	TP53	PPP1CB	EGR1	PPP3CA	YWHAE	OGT	CST3	CBX6	CAMK2D					
Endothelial cells	MYC	APP	PTGS2	ICAM1	GJA1	PIK3R3	F2R	CRK	RHOC	RHOB				
Mast cells	ICAM1	CYCS	RELB	BCL2A1	PTGS2	NFKB1	NFKB2	NFKBIB	DDX21	HSP90B1	CD69			
Cancer stem cells	TP53	MYC	EGR1	APP	PPP1CB	RHOC	GJA1	MTA2	STAT2	YWHAE	PPP3CA			
CRISPLD2^+^ cells	TP53	MYC	EP300	EGR1	CHD4	JUND	PPP1CB	FOS	HGF	YWHAE	BRD4			
Fibroblasts	APP	CST3	HSP90B1	IGFBP3	ACTN4	BSG								
Myofibroblasts	ZYX	BSG	ACTN4	PFN2	FERMT2	APP								
Smooth muscle cells	APP	FOS												
T cells	ITGB2	HNRNPA1	NFKB1	SF1	RPL27A	RBM8A	RPL14	DDX21	CCL4	RPLP2	RBM39	IL2RG	CD69	IL21R
NK cells	ITGB2	HNRNPA1	FOS	RPL27A	RPL14	RPLP2	RBM8A	CCL4	SF1	RPL38	EEF1D	RBM39	DDX21	PGK1

### Prediction of drugs for disease-related genes in single cells

**FIGURE 5:**
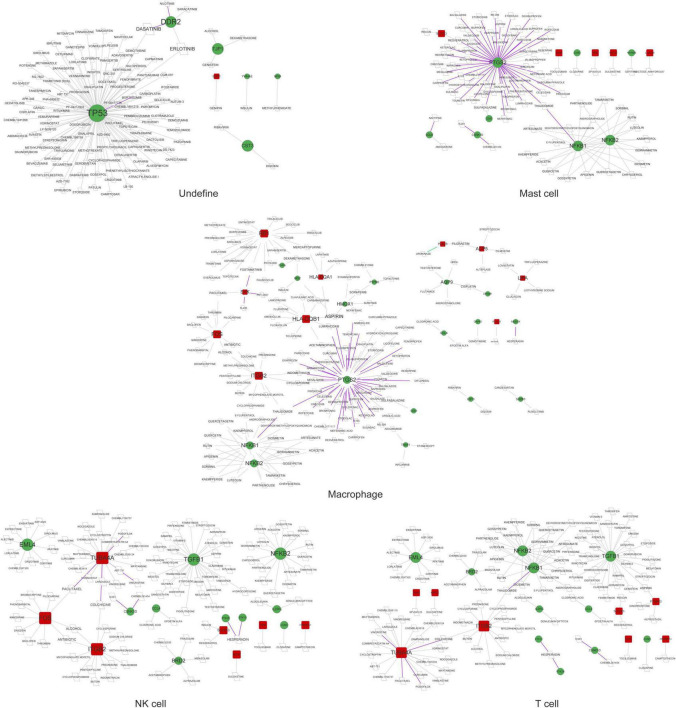
The drug–gene network in undefined cells, mast cells, macrophages, NK cells, and T cells. Red squares represent upregulated mRNAs; green circles represent downregulated mRNAs; purple lines represent the drug as an antagonist or inhibitor; green lines represent the drug as a promoter or adjuvant; gray lines represent an unknown effect.

**FIGURE 6:**
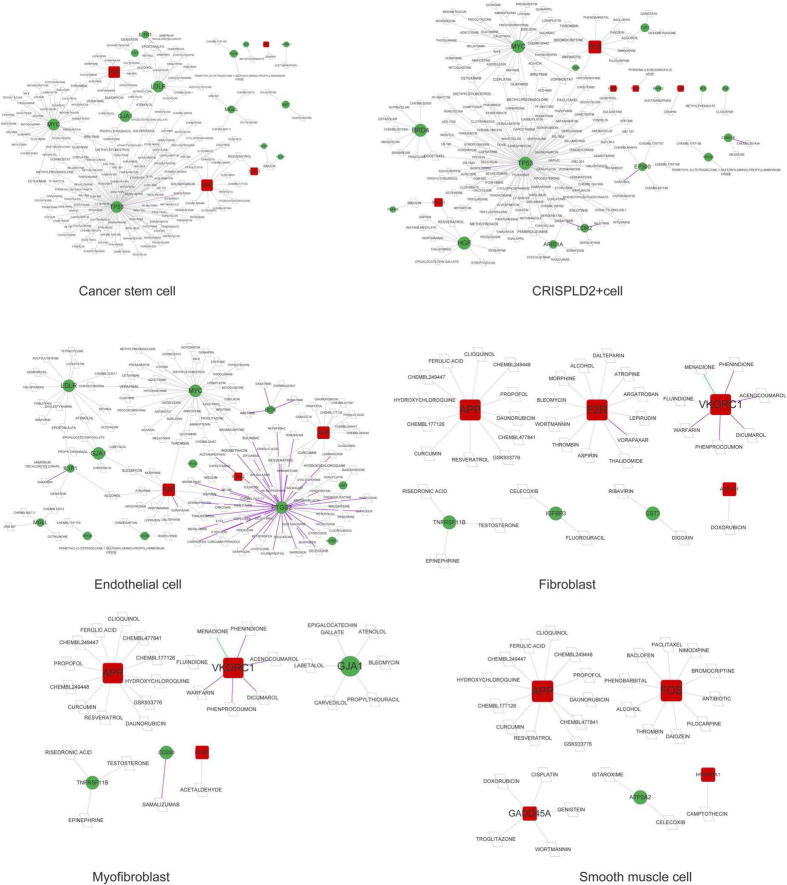
The drug–gene network in cancer stem cells, CRISPLD2^+^ cells, endothelial cells, fibroblasts, myofibroblasts, and smooth muscle cells. Red squares represent upregulated mRNAs; green circles represent downregulated mRNAs; purple lines represent the drug as an antagonist or inhibitor; green lines represent the drug as a promoter or adjuvant; gray lines represent an unknown effect.

As shown in Figures 5, 6, prostaglandin-endoperoxide synthase 2 (*PTGS2*), *TP53*, *APP*, transforming growth factor beta 1 (*TGFB1*), and *MYC* were regulated by multiple drugs, suggesting that they may serve as potential drug targets.

### Construction of the disease-related circRNA–miRNA–mRNA network in single cells

**FIGURE 7:**
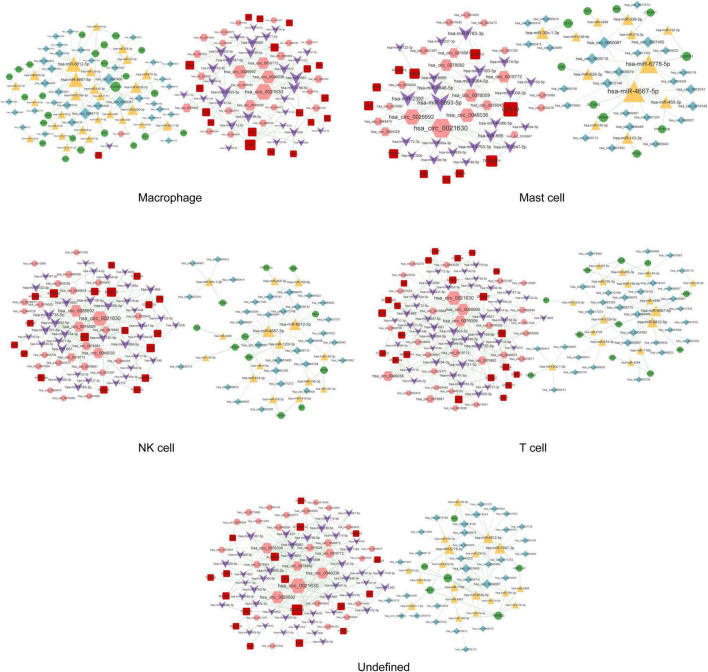
The circRNA–miRNA–mRNA regulatory network in macrophages, mast cells, NK cells, T cells, and undefined cells. Red squares represent upregulated mRNAs; green circles represent downregulated mRNAs; yellow triangles represent upregulated miRNAs; purple arrows represent downregulated miRNAs; pink hexagons represent upregulated circRNAs; blue rhombuses represent downregulated circRNAs; dotted lines indicate competitive binding of circRNAs to miRNA; solid lines indicate regulation of mRNAs by miRNAs. The node size represents the degree of connectivity.

**FIGURE 8:**
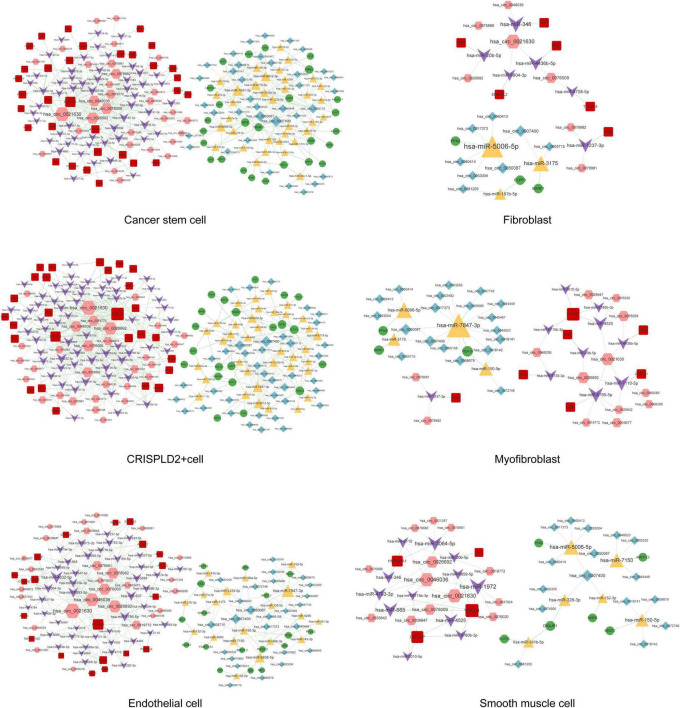
The circRNA–miRNA–mRNA regulatory network in cancer stem cells, fibroblasts, CRISPLD2^+^ cells, myofibroblasts, endothelial cells, and smooth muscle cells. Red squares represent upregulated mRNAs; green circles represent downregulated mRNAs; yellow triangles represent upregulated miRNAs; purple arrows represent downregulated miRNAs; pink hexagons represent upregulated circRNAs; blue rhombuses represent downregulated circRNAs; dotted lines indicate competitive binding of circRNAs to miRNA; solid lines indicate regulation of mRNAs by miRNAs. The node size represents the degree of connectivity.

A total of 291, 318, 262, 145, 530, 563, 21, 52, 59, 313, and 276 circRNA–miRNA–mRNA relations were identified in the macrophages, undefined cells, endothelial cells, mast cells, cancer stem cells, CRISPLD2^+^ cells, fibroblasts, myofibroblasts, smooth muscle cells, T cells, and NK cells, respectively. The constructed networks are shown in Figures 7, 8. The numbers of circRNA–miRNA–mRNA network nodes and relation pairs in each cell type are shown in Table 4.

**TABLE 4:** The numbers of nodes and relation pairs (edges) in the circRNA–miRNA–mRNA network of each cell type.

Cell type	Node				Edge		
	circRNA	miRNA	mRNA	Total	circRNA–miRNA	miRNA–mRNA	Total
Macrophages	61	57	43	161	213	78	291
Undefined cells	59	62	24	145	258	79	337
Endothelial cells	52	62	37	151	216	78	294
Mast cells	45	35	21	101	120	39	159
Cancer stem cells	66	88	58	212	322	151	473
CRISPLD2^+^ cells	68	99	48	215	375	161	536
Fibroblasts	15	9	9	33	21	9	30
Myofibroblasts	32	14	10	56	52	14	66
Smooth muscle cells	29	18	10	57	57	20	77
T cells	59	58	33	150	236	76	312
NK cells	56	51	30	137	201	69	270

### Function analysis of circRNA–miRNA–mRNA networks

**FIGURE 9:**
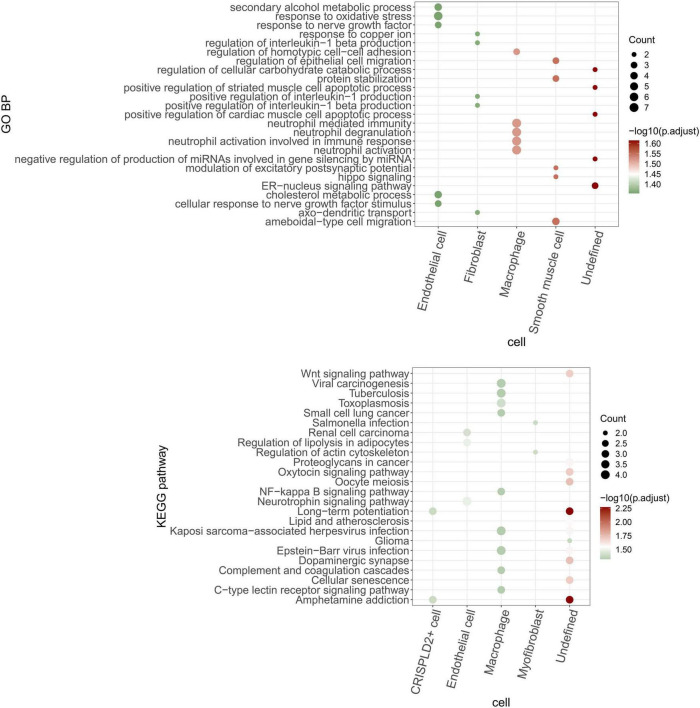
GO BP and KEGG pathways are enriched by mRNAs in the circRNA–miRNA–mRNA regulatory network of each cell type. The bubble size indicates the number of enriched genes. The color shows the enrichment significance and red is the most significant.

The function of the circRNA–miRNA–mRNA network in each cell type was analyzed based on the mRNAs in the network. As shown in Figure 9, the endothelial cells, fibroblasts, macrophages, smooth muscle cells, and undefined cells were significantly enriched in 11, 21, 5, 20, and 53 GO BP pathways, respectively. Meanwhile, the CRISPLD2^+^ cells, endothelial cells, macrophages, myofibroblasts, and undefined cells were significantly enriched in 2, 3, 9, 2, and 12 KEGG pathways, respectively.

## Discussion

Chronic thromboembolic pulmonary hypertension is a major cause of severe pulmonary hypertension (1), but the underlying molecular mechanisms remain incompletely understood. In this study, we performed both gene chip array and scRNA-seq analyses to explore the pathogenic mechanisms of CTEPH. We identified highly expressed genes in 11 cell types, and then intersection analysis with DE mRNAs was performed to obtain disease-related genes in each cell type. TP53, ICAM1, APP, ITGB2, MYC, and ZYX had the highest degrees of connectivity in the PPI networks of different cell types, suggesting that they may play important roles in the progression of CTEPH. The circRNA–miRNA–mRNA regulatory network in each cell type was then constructed to further elucidate the molecular mechanisms underlying the progression of CTEPH.

Chronic thromboembolic pulmonary hypertension is characterized by pulmonary vascular remodeling resulting from increased pulmonary arterial pressures. Fibroblasts, smooth muscle cells, endothelial cells, and myofibroblasts all play important roles in vascular remodeling (26, 27). In addition, hypertrophy caused by the increased proliferation or reduced apoptosis of vascular smooth muscle cells as well as the excessive proliferation of endothelial cells eventually results in lumen obliteration (27). APP is a type I single-pass transmembrane glycoprotein with receptor-like structural characteristics; however, its cellular function remains unclear (28, 29). A previous study has shown that the serum levels of amyloid-beta, which is produced by proteolysis of APP, are significantly increased in patients with chronic obstructive pulmonary disease with poor pulmonary function (30). Here, we found that APP was a hub node in the PPI networks of fibroblasts, smooth muscle cells, endothelial cells, and myofibroblasts. As APP can be regulated by a variety of drugs, it may serve as a potential drug target for CTEPH. *APP* was also involved in the circ_0026692-miR-20b-5p-*APP* and circ_0021630-miR-20b-5p-*APP* regulatory axes in all four cell types. Moreover, recent evidence has revealed that chronic obstructive pulmonary disease increases the risk of complications and mortality in patients with CTEPH during the early postoperative period after a pulmonary endarterectomy (31). Taken together, the above data imply that fibroblasts, smooth muscle cells, endothelial cells, and myofibroblasts may be involved in the development of CTEPH *via* the circ_0026692/circ_0021630–miR-20b-5p–*APP* regulatory axis.

Accumulating evidence has suggested that the immune system plays a key role in the pathogenesis of CTEPH (32). Moreover, increased systemic inflammation is related to local inflammatory cell infiltration in major pulmonary arteries at the advanced stage of CTEPH (7). In this study, four types of immune cells were identified. ICAM1 had the highest degree of connectivity in macrophages and mast cells, while ITGB2 had the highest degree of connectivity in T cells and NK cells. Soluble ICAM1 is present in the normal circulation, and its level is elevated in patients with endothelial activation-related disorders. Furthermore, the upregulation of ICAM1 affects the adhesion of circulating immune cells to the pulmonary endothelium, thereby promoting immune cell migration and perivascular infiltration (33). Blair et al. (34) also have reported that ICAM1 is essential for inflammatory cell recruitment in pulmonary vascular lesions in pulmonary arterial hypertension. *ITGB2* encodes an integrin beta chain involved in cell adhesion. A recent study has reported that inhibition of broad-spectrum integrin improves distal pulmonary artery remodeling, suggesting that integrin may contribute to the pathogenesis of pulmonary arterial hypertension (35). Collectively, we speculated that these immune cells might be associated with inflammatory cell recruitment in CTEPH by regulating the expression of *ICAM1* and *ITGB2*. In addition, circ_0021630 had the highest degree of connectivity in the circRNA–miRNA–mRNA regulatory networks of the four types of immune cells, indicating the regulatory potential of circ_0021630 in CTEPH-related immune responses.

TP53 had the highest degree of connectivity in the PPI networks of cancer stem cells, CRISPLD2^+^ cells, and undefined cells. *CRISPLD2* is an endogenous anti-inflammatory gene in lung fibroblasts, which can inhibit proinflammatory signaling in pulmonary epithelial cells (36). GO analysis showed that undefined cells were associated with the positive regulation of striated muscle cell apoptosis. Striated muscles, which are affected in pulmonary arterial hypertension, are associated with exercise intolerance in these patients (37). The TP53 encoding protein (p53) is a transcription factor involved in DNA repair, cell cycle arrest, and apoptosis (38). Dysregulation of p53 in pulmonary artery smooth muscle cells (PASMCs) plays an important role in vascular remodeling, a key process contributing to the progression of CTEPH (39, 40). Meanwhile, inhibition of TP53 suppresses mitochondrial respiration and induces glycolysis in PASMCs, which show a proliferative phenotype similar to that of cancer cells. Hence, CTEPH is considered a cancer-like disease in terms of PASMC remodeling (41). *TP53* is also involved in a circRNA–miRNA–mRNA regulatory network (e.g., circ_0007400–miR-6812-5p–*TP53*) in cancer stem cells and CRISPLD2^+^ cells. These data suggest that circ_0007400 may act as a competing endogenous RNA in cancer stem cells and CRISPLD2^+^ cells to promote the progression of CTEPH by regulating *TP53*.

There are some limitations in this study that must be addressed. First, we only performed bioinformatics analysis without verification experiments. Second, the sample size was relatively small. The measurement of the key circRNA and miRNA expression in tissues and studies with a large sample size are needed to validate the current findings. Thirdly, tissue samples were collected from patients undergoing pulmonary endarterectomy because it is currently the only curative treatment for CTEPH. However, it should be noted that pulmonary endarterectomy samples were derived from large vessels, while CTEPH is also related to a small vessel arteriopathy.

In conclusion, fibroblasts, smooth muscle cells, endothelial cells, and myofibroblasts may be involved in the development of CTEPH *via* the circ_0026692/circ_0021630–miR-20b-5p–*APP* regulatory axis. Additionally, macrophages, mast cells, T cells, and NK cells may be associated with inflammatory cell recruitment in CTEPH by regulating the expression of *ICAM1* and *ITGB2*. Moreover, circ_0007400 may contribute to the progression of CTEPH by acting as a competing endogenous RNA to regulate *TP53* in cancer stem cells and CRISPLD2^+^ cells. Our study, for the first time, identified the key mRNAs, miRNAs, and circRNAs, as well as their possible regulatory relations, in CTEPH using both gene chip array and scRNA-seq analyses. These data may contribute to a better understanding of the pathological mechanisms of CTEPH.

## Data availability statement

The datasets generated and analyzed during the current study are available from the corresponding authors on reasonable request.

## Ethics statement

This study was approved by the Ethics Committee of Beijing Chao-Yang Hospital, Capital Medical University (Approval numbers: 2019-K-28 and 2015-7-24-8) and performed in accordance with the principles outlined in the Declaration of Helsinki. The requirement for written informed consent was waived because discarded pulmonary endarterectomized tissues and blood samples were used in this study, while the research involved no risk to the subjects and the waiver did not adversely affect the rights and welfare of the subjects.

## Author contributions

RM, ZZ, JZ, and YY: conception and design of the research. RM, XD, JG, YL, XG, JFW, QH, and YW: acquisition of data. RM, XD, JG, YL, XG, JFW, QH, YW, JL, SY, TK, ML, and JW: analysis and interpretation of data. RM, XD, JG, YL, XG, JFW, and QH: statistical analysis. RM, YL, SY, ML, ZZ, JZ, and YY: funding acquisition. RM, XD, and JG: drafting the manuscript. RM, JW, ZZ, JZ, and YY: revision of manuscript for important intellectual content. All authors read and approved the final manuscript.

## Abbreviations

CTEPH: chronic thromboembolic pulmonary hypertension
scRNA-seq: single-cell RNA-sequencing
miRNAs: microRNAs
circRNAs: circular RNAs
PPI: protein–protein interaction
RNA-seq: RNA sequencing
DE: differentially expressed
GEO: gene expression omnibus
GO: gene ontology
BP: biological process
KEGG: Kyoto Encyclopedia of genes and genomes
NK: natural killer
ICAM1: intercellular adhesion molecule-1
APP: amyloid beta precursor protein
ITGB2: integrin subunit beta 2
MYC: MYC proto-oncogene, bHLH transcription factor
ZYX: Zyxin
PTGS2: prostaglandin-endoperoxide synthase 2
TGFB1: transforming growth factor beta 1
PASMCs: pulmonary artery smooth muscle cells.
